# Conjugative DNA Transfer From *E. coli* to Transformation-Resistant Lactobacilli

**DOI:** 10.3389/fmicb.2021.606629

**Published:** 2021-02-11

**Authors:** Sara Samperio, Dolores L. Guzmán-Herrador, Rigoberto May-Cuz, Maria Cruz Martín, Miguel A. Álvarez, Matxalen Llosa

**Affiliations:** ^1^Instituto de Biomedicina y Biotecnología de Cantabria (IBBTEC), Universidad de Cantabria-CSIC-SODERCAN, Santander, Spain; ^2^Dairy Research Institute (IPLA-CSIC), Villaviciosa, Spain

**Keywords:** bacterial conjugation, lactic acid bacteria, *Lactobacillus*, *Staphylococcus epidermidis*, plasmid RP4, plasmid R388

## Abstract

Lactic acid bacteria (LAB) belonging to the genus classically known as *Lactobacillus*, recently split into 25 different genera, include many relevant species for the food industry. The well-known properties of lactobacilli as probiotics make them an attractive model also for vaccines and therapeutic proteins delivery in humans. However, scarce tools are available to accomplish genetic modification of these organisms, and most are only suitable for laboratory strains. Here, we test bacterial conjugation as a new tool to introduce genetic modifications into many biotechnologically relevant laboratory and wild type lactobacilli. Using mobilizable shuttle plasmids from a donor *Escherichia coli* carrying either RP4 or R388 conjugative systems, we were able to get transconjugants to all tested *Lactocaseibacillus casei* strains, including many natural isolates, and to several other genera, including *Lentilactobacillus parabuchneri*, for which no transformation protocol has been reported. Transconjugants were confirmed by the presence of the *oriT* and 16S rRNA gene sequencing. Serendipitously, we also found transconjugants into researcher-contaminant *Staphylococcus epidermidis*. Conjugative DNA transfer from *E. coli* to *S. aureus* was previously described, but at very low frequencies. We have purified this recipient strain and used it in standard conjugation assays, confirming that both R388 and RP4 conjugative systems mediate mobilization of plasmids into *S. epidermidis*. This protocol could be assayed to introduce DNA into other Gram-positive microorganisms which are resistant to transformation.

## Introduction

Lactic acid bacteria (LAB) are a heterogenic group of Gram-positive bacteria with the capacity of producing lactic acid as the main product of their sugar metabolism. Consequently, LAB are an essential microbial group in the food industry due to their use as starters in the elaboration of a great variety of fermented food and drinks, being responsible for their organoleptic properties and acting as natural preservatives (Smit and Smit, 2005; Jany and Barbier, 2008; Börner et al., 2019). Due to their extensive use during the last centuries in the food industry, some species are Generally Regarded As Safe (GRAS) by the Food and Drugs Administration (FDA) and have the status of Qualified Presumption of Safety (QPS) by the European Food Safety Authority (EFSA). The genus *Lactobacillus*, in addition, contains some strains that are well-known probiotics. Up to now, the genus *Lactobacillus* was exceptionally large and diverse, as it comprised 261 species very different at the phenotypic, ecological and genotypic level. Recently, the genus has been revisited and a new classification into 25 genera has been established (Zheng et al., 2020), which helps to reflect the great biodiversity among the species that previously were grouped as *Lactobacillus*. In this work, the term “lactobacilli” will remain used to designate all organisms previously classified as *Lactobacillus* up to 2020.

The use of lactobacilli has earned interest in human and animal biomedical applications (Wells and Mercenier, 2008; Cano-Garrido et al., 2015; Wang et al., 2016). They are crucial members of the microbiota of human mucosal surfaces, where they are involved in homeostasis processes, providing protection against pathogenic bacteria and stimulating the immune system (Isolauri and Ouwehand, 2004; Bernardeau et al., 2008). Lactobacilli have been proposed as ideal live vectors for the *in situ* production of therapeutic agents in the oral, nasal, genital and intestinal mucosae (Cano-Garrido et al., 2015; Wang et al., 2016; Rio et al., 2019), due to their tolerance to temperature, low pH, bile salts, or high alcohol concentrations (Bosma et al., 2017). So far, lactobacilli have been used as adjuvants or prophylactic agents against many different diseases (Reid, 2017; Mays and Nair, 2018) as well as in a range of animal husbandries (Syngai et al., 2016). Furthermore, their use in therapeutics for prevention and diagnosis (Mays and Nair, 2018) is gaining attention. However, the extended use of lactobacilli in industrial and biomedical applications is limited, since genetic tools are still underdeveloped, especially for wild-type strains (Bosma et al., 2017).

LAB were a pioneer group studied for development of genetic tools (de Vos, 2011), but these efforts were mainly focused on obtaining food-grade microorganisms rather than optimizing mutagenesis procedures (Derkx et al., 2014; Bachmann et al., 2015; Johansen, 2018; Vida and Berlec, 2020). There are several targeted genome editing methods currently available for LAB (Martin et al., 2000; Bosma et al., 2017; Hatti-Kaul et al., 2018). First studies focused on *Lactococcus lactis* and *Lactiplantibacillus plantarum*, due to their importance as starter cultures and probiotics, respectively, but several other LAB species have been found to be susceptible to genetic modification albeit with significantly lower efficiencies (de Vos, 2011; Bosma et al., 2017). The first step to accomplish targeted genetic modification is the introduction of DNA, which can be challenging in Gram-positive bacteria due to the thick peptidoglycan layer in their cell wall. The most widely used method is electroporation. Although a wide range of LAB species have been successfully transformed using generalized electroporation protocols, efficiencies varied strongly among strains and protocols need to be optimized (Landete et al., 2014; Bosma et al., 2017). In particular, transformation of lactobacilli wild-type strains has proven difficult or even not feasible. Thus the importance of exploring new approaches for efficient DNA introduction in these important LAB.

Bacterial conjugation is an efficient mechanism of horizontal gene transfer (HGT) of DNA from a donor bacterium to a recipient one which requires physical contact between them. Bacterial conjugation confers a high genomic plasticity to the prokaryotic world (de la Cruz and Davies, 2000), being the most important means of spreading resistance and virulence factors among bacteria. The conjugative machinery is composed by a Type IV secretion system (T4SS), which constitutes the physical channel for secretion of the DNA, and a number of proteins which recognize and process the DNA to be transferred (Cabezón et al., 2015). Among them, a key enzyme is the relaxase, which attaches covalently to the DNA and pilots it into the recipient cell (Guzmán-Herrador and Llosa, 2019). The DNA to be transferred must have a sequence of 100–400 bp known as origin of transfer (*oriT)*, which is recognized by the relaxase, where it binds and cleaves the strand to be transferred.

Conjugation has been described in both Gram-negative and -positive bacteria, and even between both bacterial groups. Conjugative transfer using the RP4 transfer system from *E. coli* to several Gram-positive bacteria was described long ago (Trieu-Cuot et al., 1987). Dominguez and Sullivan (2013) describe a robust conjugation protocol that can be used in the transfer of genetic material from *E. coli* to several *Bifidobacterium* species. Although conjugative plasmids and transposons are very common in LAB, the details of conjugative mechanisms are still under research (Kullen and Klaenhammer, 2000; Bron et al., 2019). It has also been described that conjugative transfer happens *in vivo* in our microbiota, including LAB species as recipients (Aviv et al., 2016). Conjugative DNA transfer from lactobacilli has been described in a few instances to other LAB, such as *Enterococcus* and *Lactococcus* (Gevers et al., 2003). However, up to date, transfer of DNA into lactobacilli by conjugation has not been reported.

The development of genetic modification tools for Gram-positive bacteria has also focused on species with clinical relevance. An example of this is illustrated by the genus *Staphylococcus*, which includes many relevant strains for human health, and is also reluctant to genetic manipulation. Staphylococci are one of the main causative agents of severe nosocomial infections which require prolonged hospitalizations (Becker et al., 2014). The majority of genetic tools have been developed for *Staphylococcus aureus*; in other staphylococci, genetic modification is often halted by the absence of efficient transformable protocols. This is the case of coagulase-negative staphylococci (Becker et al., 2014) which include species with increasing interest in human health, such as the emerging human pathogen *Staphylococcus epidermidis* (Otto, 2009). Up to now, staphylococcal species are transformed via electroporation or, less frequently, by protoplast transformation (Götz and Schumacher, 1987; Augustin and Götz, 1990; Löfblom et al., 2007). However, the restriction-modification systems present in *S. aureus* truncate the uptake of foreign DNA (Waldron and Lindsay, 2006; Xu et al., 2011; Monk and Foster, 2012; Monk et al., 2012). The use of *E. coli* strains lacking *dcm* for production of unmethylated DNA allowed electroporation of particular strains (Monk et al., 2012; Costa et al., 2017), but it requires large amounts of DNA and is limited to specific isolates. Transformation is especially inefficient for *S. epidermidis* strains (Costa et al., 2017). Bacterial conjugation from *E. coli* to *S. aureus* was initially reported, albeit at low frequency (Trieu-Cuot et al., 1987), and no follow-up works are available.

The limitations of current electroporation protocols for the introduction of DNA, especially in wild-type strains, prompted us to assay conjugation as an alternative to transfer DNA into lactobacilli. Furthermore, conjugation is considered a natural mechanisms and therefore is a more accepted approach than electro-transformation (Pedersen et al., 2005). To this end, we have optimized a conjugation protocol from *E. coli* to lactobacilli using the promiscuous conjugative plasmids R388 and RP4. Using this protocol, we obtained transconjugants in a number of genera and species, including many wild-type strains. Serendipitously, we found that this conjugation protocol also mediates conjugal transfer from *E. coli* to *S. epidermidis*, a researcher-contaminant bacterium which normally colonizes the human skin. This conjugation protocol could be a useful approach to genetically modify other Gram-positive microorganisms which are resistant to electroporation.

## Materials and Methods

### Bacterial Strains and Culture Conditions

The bacterial strains used in this study are listed in Table 1. *Escherichia* coli strains were grown at 37°C in LB media and when necessary supplemented with 100 μg/ml ampicillin (Ap), or 50 μg/ml kanamycin (Km). Lactobacilli and *S. epidermidis* were grown at 37°C without aeration in MRS medium (Oxoid, Basingstoke, Hampshire, England) or on solid MRS plates supplemented with 2% agar, supplemented with 5 μg/ml erythromycin (Em) when indicated.

**TABLE 1:** Bacterial strains used in the present study.

**Bacteria**	**Relevant properties^a^**	**Reference or source**
*Escherichia coli* DH5α	Nx^*R*^; *F- endA1 hsdR17 supE44 thi-1 recA1 gyrA96 relA1*Δ*(argF-lacZYA) U 169*Φ*80dlac*Δ*M15*	Grant et al., 1990
*E. coli* D1210	Sm^*R*^*; recA hspR hsdM rpsl lacI^*q*^*	Sadler et al., 1980
*E. coli* S17.1	Sm^*R*^; (F-)RP4-2-Tc:Mu *aph*:Tn*7 recA*	Simon et al., 1983
*Furfurilactobacillus rossiae* D87	Isolated from bread dough	Del Rio et al., 2018
*Lacticaseibacillus casei* 393	Laboratory strain	Hansen and Lessel, 1971
*L. casei* 12003	Isolated from wheat dough—white bread	Alvarez-Sieiro et al., 2016
*L. casei* 12032	Isolated from wheat dough—Pasta	Alvarez-Sieiro et al., 2016
*L. casei* 12042	Isolated from wheat dough—white bread	Alvarez-Sieiro et al., 2016
*L. casei* 13b	Dairy-derived—zamorano	Herrero-Fresno et al., 2012
*L. casei* 41b	Dairy-derived—zamorano	Herrero-Fresno et al., 2012
*L. casei* 5b	Dairy-derived—zamorano	Herrero-Fresno et al., 2012
*L. casei* 61b	Dairy-derived—cabrales	Herrero-Fresno et al., 2012
*L. casei* E2	Dairy-derived—emmental	Herrero-Fresno et al., 2012
*Lacticaseibacillus paracasei* 1D*-*CCC76	Isolated from cheese	IPLA collection
*Lactiplantibacillus plantarum* IPLA88	Isolated from bread dough	Laredo et al., 2013
*Lactobacillus crispatus* HFS47	Isolated from human feces	IPLA collection
*Lactobacillus gasseri* HFS29	Isolated from human feces	IPLA collection
*Latilactobacillus curvatus* 1b*-*VPZ3	Isolated from cheese	IPLA collection
*Lentilactobacillus buchneri* 1D*-*VPC30	Isolated from cheese	IPLA collection
*Lentilactobacillus parabuchneri* 11122	Dairy-derived—emmental	Diaz et al., 2016
*Levilactobacillus brevis* 1D-VCC39	Isolated from cheese	IPLA collection
*Ligilactobacillus ruminis* HFS44	Isolated from human feces	IPLA collection
*Limosilactobacillus reuteri* IPLA11078	Isolated from cheese	IPLA collection
*Limosilactobacillus vaginalis* IPLA11050	Isolated from cheese	Diaz et al., 2015a
*Loigolactobacillus coryniformis* MZ25	Isolated from cheese	IPLA collection
*Staphylococcus epidermidis*	Human skin—spontaneous isolate	This work

*^a^Nx^*R*^, nalidixic acid resistance; Sm^*R*^, streptomycin resistance.*

The *S. epidermidis* strain used in this work was isolated from the researcher’s skin. The hands were placed on MRS-agar plates without antibiotics, which were then incubated at 37°C. The colonies grown were replicated on MRS-agar with and without Em 5 μg/ml. Em-sensitive colonies were selected, their 16S rRNA gene sequence was amplified by PCR, and DNA sequence was determined (STABVIDA) to confirm they were *S. epidermidis*.

### DNA Manipulation

In order to extract genomic DNA from lactobacilli and *S. epidermidis*, a colony from an MRS-agar plate is punctured and resuspended in 50 μl of TE buffer. 50 μl of chloroform are added and mixed thoroughly until the mixture is homogeneous. The mixture is then centrifuged 10 min at 4°C and three phases appear. The top phase containing the genomic DNA is collected carefully and used directly for PCR analysis.

Plasmid DNA was isolated from *E. coli* with the GenElute Plasmid Miniprep Kit (Sigma). From lactobacilli and *S. epidermidis*, the protocol of Anderson and Mckay (1983) was followed with modifications to lyse the cells previous to plasmid DNA purification with the GenElute Plasmid Miniprep Kit, as follows. *Lactobacillus* and *S. epidermidis* strains were grown overnight in MRS supplemented with Em (5 μg/ml) for plasmid selection. Two milliliter cultures were centrifuged 10 min at 14,000 rpm. The pellet was resuspended in STE (sucrose 10.3%, Tris HCl 25 mM pH8, EDTA 10 mM) and centrifuged again 10 min at the same speed. The pellet was frozen at −80°C for 15 min. Then, the pellet was resuspended in 200 μl of lysis buffer (sucrose 20%, Tris HCl 10 mM pH8, EDTA 10 mM, NaCl 10 mM) with lysozyme (30 mg/ml), 2 μl of RNAse (25 mg/ml) and 20 μl of proteinase K (20 mg/ml). The sample was homogenized by vortexing, and incubated at 55°C during 30 min. Then, the lysates were applied to GenElute Plasmid Miniprep Kit (Sigma-Aldrich) to purify the plasmid DNA following the manufacturer’s protocol.

DNA and PCR products were visualized by agarose gel electrophoresis stained with SYBR Safe (Invitrogen) and visualized with a Gel Doc2000 UV system, and images were analyzed with Quantity One software (BioRad). HyperLader I (Biolabs) was used as a molecular weight marker. DNA was quantified using a Nano-Drop Spectrophotometer ND-1000. GenElute PCR Clean-Up Kit (Sigma) was used for purification of PCR products, and GenElute Agarose Spin Columns (Sigma) were used for DNA purification from agarose gels.

### Plasmids and Plasmid Constructions

Plasmids used in this work and their relevant properties are listed in Table 2. Plasmid constructions were done by standard recombinant DNA techniques. Plasmid pEM110 was digested with the enzymes *Cla*I and *Sma*I (Thermo Fisher Scientific). The *oriT* sequences of plasmids RP4 and R388 were PCR-amplified from plasmids pLA31 and pLA32, respectively, using the oligonucleotides shown in Table 3 and high fidelity DNA polymerase PCRBIO HiFi (PCRBIOSYSTEMS). PCR fragments were digested with the same enzymes and ligated with the vector. Ligations were electroporated into *E. coli* (see below). The DNA sequence of the inserts was determined (STABVIDA) to verify the correct assembly of the new plasmids.

**TABLE 2:** Plasmids used in the present study.

**Plasmid**	**Relevant properties^a^**	**Reference or source**
pCOR48	pEM110- based, shuttle vector *E. coli* –*Lactobacillus*, Ap^*R*^ Em^*R*^; R388 *oriT*	This work
pCOR49	pEM110-borned, shuttle vector *E. coli* –*Lactobacillus*, Ap^*R*^ Em^*R*^; RP4 *oriT*	This work
pEM110	P8014-2 *oriV* (*L. plantarum*), pBR322 *oriV* (*E. coli*), Em^*R*^	Martín et al., 2004
pLA31	pSU36:RP4 *oriT*	Agúndez et al., 2012
pLA32	pSU36:R388 *oriT*	
pSU711	Km^*R*^; R388 Δ*oriT*	Demarre et al., 2004

*^a^Ap^*R*^, ampicillin resistance; Cm^*R*^, chloramphenicol resistance; Em^*R*^, erythromicyn resistance; Km^*R*^, kanamycin resistance.*

**TABLE 3:** Primers used for PCR.

**Purpose of PCR**	**Sequence**
***oriT* for cloning**	
*Cla*I-*oriT* R388-1	5′-CCGACTATCGATTCTCATTTTCTGCATCATGGTAG-3′
*oriT* R388 401-*Sma*I	5′-AGCTATCCCGGGCCGCCTCGTCCTCCAAAA-3′
*ClaI-oriT RP4-1*	5′-CCGACTATCGATCCGCTTGCCCTCATCTG-3′
*oriT* RP4-*Sma*I	5′-AGCTTTCCCGGGCGCTTTTCCGCTGCATAA-3′
**Transconjugant confirmation**	
*oriT* R388 F	5′-CCAAGTCTACTCATTTTCTGCATCATTGT-3′
*oriT* R388 R	5′-CCAAGTCTACCTCTCCCGTAGTGTTACT-3′
*ClaI-oriT RP4-1*	5′-CCGACTATCGATCCGCTTGCCCTCATCTG-3′
*oriT* RP4-*Sma*I	5′-AGCTTTCCCGGGCGCTTTTCCGCTGCATAA-3′
**16S rRNA determination**	
16S 1492R	5′-TACGGYTACCTTGTTACGACTT-3′
16S 27F	5′-AGAGTTTGATYMTGGCTCAG-3′

*Underlined sequences represent the restriction sites.*

### *E. coli* Electroporation

For preparation of electrocompetent cells, bacteria were grown to OD600 = 0.5–0.6, and pelleted by centrifugation at 4°C. Two series of washes and centrifugations (6,000 rpm on a Beckman JA-10 rotor) of 1vol milliQ water and a final wash in 1/50 volume 10% glycerol at 4°C were applied. Cells were resuspended in 1/500 vol 10% glycerol and aliquoted in 50 μl samples. Aliquots were frozen on dry ice and kept at −70°C until usage. Aliquots were mixed with < 10 ng of DNA in a 0.2 cm Gene Pulser^®^ cuvette (BioRad) and subjected to an electric pulse (2.5 kV, 25 μF and 200 Ω) in a MicroPulser^TM^ (BioRad). Electroporated cells were added to 1 ml LB and incubated with shaking at 37°C to allow antibiotic expression. After incubation cells were plated on antibiotic containing media.

### Bacterial Conjugation

Standard conjugation assays in *E. coli* were performed as described in Grandoso et al. (2000). The conjugation protocol from *E. coli* to lactobacilli was optimized starting from the one previously described. Once the new protocol was established, conjugation to lactobacilli was performed as follows: First, donor *E. coli* strains were grown on LB supplemented with antibiotics overnight. Recipient lactobacilli were grown on MRS without antibiotics. The matings were performed on solid media by mixing the same volume of donor and recipient strains (100 μl of the overnight cultures) after washing with BHI media (Oxoid, Basingstoke, Hampshire, England). The bacterial mixture was then washed with BHI again, resuspended in 20 μL of BHI and transferred to a conjugation filter (0.22 μm nitrocellulose, Millipore) on a BHI-agar plate. The mating mixtures were incubated at 37°C for 24 h. Then, the filter was resuspended on BHI and appropriate dilutions were made and plated on selective media for donors, recipients and transconjugants. Donor *E. coli* were plated on LB agar with antibiotics for strain and plasmids selection. Recipient strains were plated on MRS. Transconjugants were plated on MRS with Em 5 μg/ml. The frequency of conjugation is expressed as the number of transconjugants per donor cell. Conjugation from *E. coli* into *Staphylococcus epidermidis* strains was performed as explained above for lactobacilli. All the manipulations of these conjugations were performed on a Faster BH-EN 2004 Class II Microbiological Safety Cabinet and using filter tips.

### Analysis of Transconjugants

Transconjugants were analyzed directly from the plate for the presence of *oriT*. PCR reactions included an extra boiling step at the beginning to break the cells. PCRs were performed using DNA polymerase KapaTaq (KapaBiosystems) and primers indicated in Table 3. PCR products were run on agarose gels to observe the expected amplification bands.

Several transconjugants from each conjugation assay were analyzed to confirm the lactobacilli or *S. epidermidis* species by PCR-amplification of the 16S rRNA gene, using the universal primer pair 27F and 1492R (Table 3; Lane, 1991), and determination of the DNA sequence from the amplicon, as explained in Diaz et al. (2016).

In order to confirm the presence of the autonomous shuttle plasmid in *S. epidermidis* transconjugants, plasmid DNA was extracted from both the transconjugants and the strain with no plasmid, and from *L. casei* with and without plasmid as a control. Plasmid DNA was visualized on agarose gels. Subsequently, this plasmid DNA was electroporated into *E. coli*, plasmid DNA extracted again from the transformants, and analyzed by restriction digestion to test its integrity.

## Results

### Bacterial Conjugation From *E. coli* to *Lacticaseibacillus casei*

In order to set up a protocol for conjugative DNA transfer into lactobacilli from laboratory *E. coli* strains, we adapted the protocol routinely used for conjugative DNA transfer among Gram-negative bacteria on solid media (Grandoso et al., 2000). We tested two well characterized conjugative systems; those of plasmids R388 and RP4, which have been previously shown to mediate conjugative DNA transfer into a broad range of recipient cells (see section “Introduction”).

For a DNA molecule to be transferred by conjugation, the only element required in *cis* is the *oriT*. We constructed mobilizable shuttle vectors carrying replication and antibiotic resistance genes for selection in *E. coli* and *Lactobacillus*, plus the *oriT* of either R388 or RP4 (pCOR48 and pCOR49; Table 2). The rest of the conjugative machinery was provided in *trans*, either using *E. coli* S17.1 strain as a donor, which has the conjugative machinery of RP4 integrated into the bacterial chromosome, or using a non-mobilizable helper plasmid which provides the R388 conjugative system (pSU711; Table 2). These plasmids were tested in conjugation between *E. coli* strains to verify their functionality (Table 4, top rows). As negative controls, we used donors harboring the mobilizable plasmids but devoid of the rest of the conjugative machinery.

**TABLE 4:** Conjugation frequency of mobilizable shuttle plasmids into *E. coli* and *Lacticaseibacillus casei 393.*

**Recipient**	**Donor strain**	**Conjugative system**	**Conjugation frequency**	**Stand. Dev.**	**n**
*E. coli* DH5α	**D1210 + pSU711 + pCOR48**	R388+	**2.5 × 10^–3^**	±1.9 × 10**^–^**^3^	3
*E. coli* DH5α	**D1210 + pCOR48**	R388−	<2.6 × 10**^–^**^7^	±1.5 × 10**^–^**^7^	3
*E. coli* DH5α	**S17.1 + pCOR49**	RP4+	**1.4 × 10^–2^**	±1.3 × 10**^–^**^2^	3
*E. coli* DH5α	**D1210 + pCOR49**	RP4−	<4.6 × 10**^–^**^6^	±4.0 × 10**^–^**^6^	3
*L. casei* 393	**D1210 + pSU711 + pCOR48**	R388+	**2.1 × 10^–4^**	±6.1 × 10**^–^**^4^	18
*L. casei* 393	**D1210 + pCOR48**	R388−	<2.0 × 10**^–^**^6^	±2.3 × 10**^–^**^6^	18
*L. casei* 393	**S17.1 + pCOR49**	RP4+	**1.8 × 10^–3^**	±2.0 × 10**^–^**^3^	8
*L. casei* 393	**D1210 + pCOR49**	RP4−	<3.0 × 10**^–^**^6^	±3.1 × 10**^–^**^6^	8

*Positive results are highlighted in bold.*

In order to optimize a new protocol for conjugation from *E. coli* to lactobacilli, we chose the laboratory strain *L. casei* 393, which is easy to grow, manipulate and transform (Chassy and Flickinger, 1987). Different conditions were tested, such as the mating time, donor/recipient cell ratio, growth phases in the bacterial cultures, and culture media in the conjugation plate. After several trials, a functional protocol for conjugation between *E. coli* and *L. casei* was established. The protocol is detailed in section “Materials and Methods.” In summary, overnight cultures of both donor and recipient bacteria were mixed on BHI medium, where both donor and recipient cells can thrive, while LB and MRS allow growth only of *E. coli* and *L. casei*, respectively, allowing counter selection of donors or recipients. The mating mixtures were incubated on solid media for 24 h. Conjugation frequencies obtained are shown in Table 4 (lower rows). Transconjugants were obtained using both R388 and RP4 conjugative systems, with frequencies only 1−log lower than between *E. coli* strains (2.1 × 10^–4^ vs. 2.5 × 10^–3^ transconjugants/donor for R388, and 1.8 × 10^–3^ vs. 1.4 × 10^–2^ for RP4).

**FIGURE 1:**
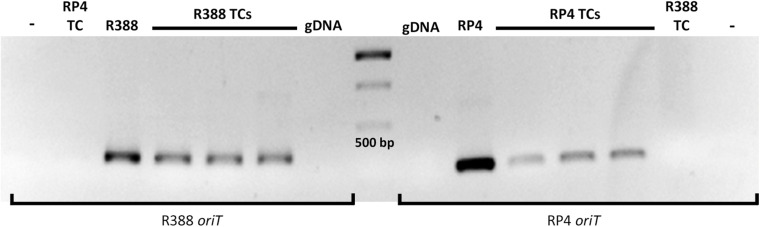
PCR analysis of *L. casei* 393 transconjugants for the amplification of R388 and RP4 *oriT*. The *oriT* amplified by PCR is indicated at the bottom (oligonucleotides indicated in Table 3). The template is indicated on top of the gel, and it was either purified DNA (R388 and RP4 as positive controls, and the recipient *L. casei* gDNA as negative control) or transconjugant (TC) colonies directly used for the PCR reactions. The expected plasmid in the transconjugants (R388 or RP4) is indicated. –, control reaction with no template DNA. The central lane is a molecular weight marker (GeneRuler 1 kb DNA ladder). The 500 bp band is indicated. Expected fragment size: 330 bp for R388 *oriT* and 280 bp for RP4 *oriT*.

Several transconjugants were selected for further analysis. Total DNA was extracted and used as a template for two PCR amplifications: (i) the 16S rRNA gene region, which was used for DNA sequence determination and confirmation that they were *L. casei*; and (ii), the corresponding *oriT*. An example of this analysis is shown in Figure 1. It can be observed that neither *oriT* is amplified from gDNA of *L. casei*, while the expected band for each *oriT* is present in DNA from the transconjugants. All these results confirm that the transconjugants obtained were *bona-fide L. casei* colonies which had received the pCOR shuttle plasmid by conjugation.

The ability to transform lactobacilli by electroporation varies widely depending not only on the genus or species, but also on the strains. Some of the more interesting strains for biotechnological purposes are wild-type isolates, which typically show lower transformation rates than laboratory strains. We tested different strains of *L. casei* as conjugation recipients, isolated from natural environments. The results (Table 5) showed that we obtained transconjugants into all strains using RP4 conjugative system, whereas R388 conjugative system provided transconjugants in a subset of strains only. Transconjugants were confirmed by PCR amplification of the *oriT*. We confirmed that conjugation frequencies varied significantly among strains of *L. casei*, and frequencies were in all cases lower than that of the laboratory strain: in the case of R388, frequencies ranged around 10^–7^–10^–5^ transconjugants/donor (compared to 10^–4^ for *L. casei* 393), and in the case of RP4, we obtained between 10^–7^ and 10^–4^ transconjugants per donor (compared to 10^–3^ for the laboratory strain).

**TABLE 5:** Conjugation frequencies from *E. coli* to different *L. casei* strains using R388 and RP4 systems^a^.

	**Conjugation frequencies**			
	**R388**		**RP4**	
**Recipient**				
**strain**	**+**	**−**	**+**	**−**
*L. casei* 393	**2.1 × 10^–4^**	<2.0 × 10**^–^**^6^	**1.8 × 10^–3^**	<3.0 × 10**^–^**^6^
*L. casei* 5 b	**3.5 × 10^–6^**	<3.6 × 10**^–^**^6^	**1.9 × 10^–4^**	<2.5 × 10**^–^**^6^
*L. casei* 13 b	**2.8 × 10^–6^**	<8.4 × 10**^–^**^6^	**3.9 × 10^–5^**	<6.0 × 10**^–^**^6^
*L. casei* E2	**1.5 × 10^–7^**	<6.6 × 10**^–^**^7^	**1.6 × 10^–4^**	<8.1 × 10**^–^**^7^
*L. casei* 41 b	< 1.3 × 10^–6^	<2.9 × 10**^–^**^6^	**1.1 × 10^–5^**	<3.0 × 10**^–^**^6^
*L. casei* 61 b	**5.0 × 10^–5^**	<5.8 × 10**^–^**^6^	**3.8 × 10^–5^**	<1.2 × 10**^–^**^5^
*L. casei* 12003	**2.4 × 10^–5^**	<1.0 × 10**^–^**^7^	**1.1 × 10^–4^**	<1.0 × 10**^–^**^7^
*L. casei* 12032	< 1.6 × 10**^–^**^6^	<1.1 × 10**^–^**^7^	**7.8 × 10^–6^**	<1.0 × 10**^–^**^7^
*L. casei* 12042	< 3.9 × 10**^–^**^8^	<2.1 × 10**^–^**^8^	**5.6 × 10^–7^**	<3.6 × 10**^–^**^7^

*^*a*^Donor strains as shown inTable 4. Data shown are the mean of 2 independent experiments. Positive results are highlighted in bold.*

### Conjugation From *E. coli* to Other Lactobacilli

The next step was to test conjugation to other wild-type lactobacilli, some of which are reluctant to genetic transformation by electroporation. Conjugation was performed using the same donor strains harboring R388 and RP4 conjugative systems as shown in Table 4, and using as positive control for conjugation *L. casei* 393 as a recipient. The conjugation frequencies obtained are shown in Table 6. Transconjugants were obtained for *Lactiplantibacillus plantarum, Lentilactobacillus buchneri, Lentilactobacillus parabuchneri, Levilactobacillus brevis*, and *Limosilactobacillus vaginalis* when using the RP4 conjugative system. No transconjugants were obtained when using the R388 conjugative system. The frequencies obtained were in all cases significantly lower than into the laboratory strain *L. casei* 393, ranging around 10^–5^–10^–6^ transconjugants per donor (vs. 10^–3^ for the laboratory strain).

**TABLE 6:** Conjugation frequencies from *E. coli* to different lactobacilli using R388 and RP4 systems^a^.

	**Conjugative system**			
	**R388**		**RP4**	
**Recipient**				
**lactobacilli**	**+**	**−**	**+**	**−**
*L. casei* 393	**2.1 × 10^–4^**	< 2.0 × 10**^–^**^6^	**1.8 × 10^–3^**	< 3.0 × 10**^–^**^6^
*L. curvatus*	<2.2 × 10**^–^**^9^	<5.7 × 10**^–^**^9^	<1.0 × 10**^–^**^7^	<1.0 × 10**^–^**^8^
*L. buchneri*	<4.8 × 10**^–^**^8^	<8.7 × 10**^–^**^8^	**6.6 × 10^–7^**	<1.1 × 10**^–^**^7^
*L. brevis*	<6.8 × 10**^–^**^7^	<2.1 × 10**^–^**^7^	**6.0 × 10^–6^**	<1.3 × 10**^–^**^6^
*L. paracasei*	<3.2 × 10**^–^**^7^	<1.4 × 10**^–^**^7^	<3.1 × 10**^–^**^6^	<1.4 × 10**^–^**^6^
*L. coryniformis*	<8.2 × 10**^–^**^7^	<1.7 × 10**^–^**^7^	<1.8 × 10**^–^**^6^	<1.6 × 10**^–^**^7^
*L. parabuchneri*	<3.9 × 10**^–^**^7^	<9.7 × 10**^–^**^8^	**2.0 × 10^–6^**	< 2.0 × 10**^–^**^6^
*L. reuteri*	<1.6 × 10**^–^**^7^	<9.1 × 10**^–^**^9^	<1.4 × 10**^–^**^7^	<6.8 × 10**^–^**^9^
*L. vaginalis*	<5.7 × 10**^–^**^7^	<9.5 × 10**^–^**^7^	**1.1 × 10^–6^**	<1.47 × 10**^–^**^7^
*L. rossiae*	<1.4 × 10**^–^**^6^	<2.0 × 10**^–^**^6^	<5.0 × 10**^–^**^6^	<1.6 × 10**^–^**^6^
*L. plantarum*	<2.6 × 10**^–^**^6^	<2.3 × 10**^–^**^8^	**1.1 × 10^–5^**	<9.0 × 10**^–^**^9^
*L. crispatus*	<1.0 × 10**^–^**^7^	<1.8 × 10**^–^**^7^	<2.0 × 10**^–^**^7^	<2.7 × 10**^–^**^7^

*^*a*^Donor strains as shown inTable 4. Data shown are the mean of 2 independent experiments. Positive results are highlighted in bold.*

**FIGURE 2:**
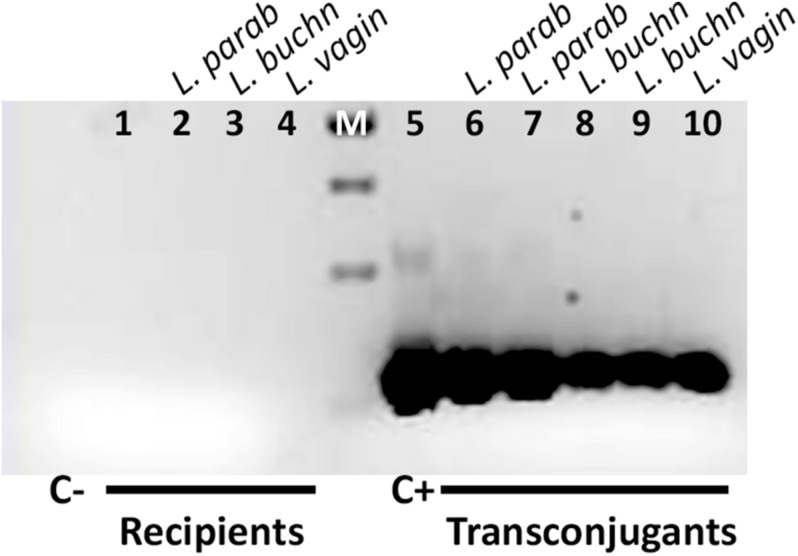
Amplification of RP4 *oriT* from transconjugants. Primers used are shown in Table 3. The template DNAs are: Lane 1: no DNA (C–, negative control). Lanes 2–4: gDNAs from recipient *L. parabuchneri, L. buchneri* and *L. vaginalis.* M, molecular marker (GeneRuler 1 kb DNA ladder). The size of the bands is 1,000, 750, 500, and 250 bp from top to bottom. The expected size of the amplicon is 280 bp. To the right, Lane 5: pCOR49 plasmid DNA (C+, positive control). Lanes 6 and 7: DNA extracts from *L. parabuchneri* transconjugants. Lanes 8 and 9: DNA extracts from *L. buchneri* transconjugants. Lane 10: gDNA extracts from *L. vaginalis* transconjugant.

Transconjugants were confirmed by the presence of the corresponding *oriT* (Figure 2). Their 16S rRNA gene sequence was amplified with primers shown in Table 3 and the DNA sequence determined, confirming in all cases the expected genera.

### Conjugation From *E. coli* to *S. epidermidis*

Serendipitously, we found a high number of putative transconjugants in one of the matings described above using the R388 conjugative system, which did not match the lactobacilli phenotype, although they did show amplification of the R388 *oriT*. Upon sequencing of the 16S rRNA gene, we found out these colonies corresponded to *Staphylococcus epidermidis*, a common isolate in human epidermis, and thus probably originated from a contamination from the researcher skin. Since this fact suggested that conjugation from *E. coli* occurred to other Gram-positives, and *S. epidermidis* itself is a recalcitrant organism of high biomedical interest, we decided to confirm and quantify this phenomenon. To confirm and quantify this finding, as well as to rule out that the observed phenotype was restricted to a particular isolate, *S. epidermidis* was isolated placing on MRS plates the hands of two other researchers, from the same and different laboratories. Colonies resembling staphylococci were obtained, cultured on MRS, checked for their sensitivity to erythromycin by replica-plating, and confirmed as *S. epidermidis* by 16S rRNA gene sequencing. A PCR for the R388 and RP4 *oriT* was performed on total DNA to verify that there was no amplification from the strains (not shown). These new isolates of *S. epidermidis* (isolates 1 and 2) were used as a recipient strains in conjugation assays from *E. coli*.

Conjugation frequencies obtained are summarized in Table 7. We obtained *S. epidermidis* transconjugants for both recipient strains, using both R388 and RP4 conjugative systems, confirming that plasmids can be mobilized by conjugation from *E. coli* to *S. epidermidis*. It is interesting to note that in this case, the R388 system was similar or even more efficient in rendering transconjugants than RP4. Transconjugants were confirmed by PCR for amplification of the corresponding *oriT*, and their 16S rRNA gene sequence was amplified by PCR and the DNA sequence determined, to verify that they were *S. epidermidis*.

**TABLE 7:** Conjugation frequencies from *E. coli* to *S. epidermidis* using conjugative systems R388 and RP4^a^.

	**Conjugation frequencies**			
	**R388**		**RP4**	
**Recipient**				
**bacteria**	**+**	**−**	**+**	**−**
*S. epidermidis* isolate 1	**2.5 × 10^–6^**	<1.4 × 10**^–^**^6^	**8.1 × 10^–8^**	<1.1 × 10**^–^**^8^
*S. epidermidis* isolate 2	**3.5 × 10^–7^**	<7.8 × 10**^–^**^7^	**1.7 × 10^–7^**	<2.1 × 10**^–^**^7^

*^*a*^Donor strains as shown inTable 4. Data shown are the mean of 3–4 independent experiments. Positive results are highlighted in bold.*

**FIGURE 3:**
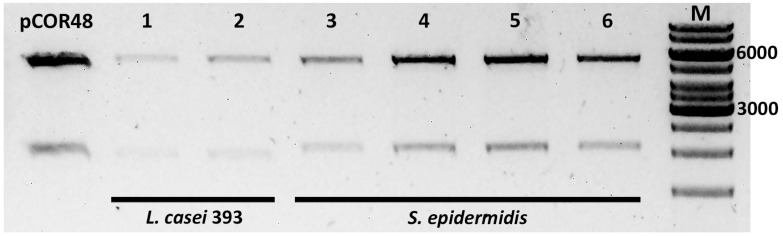
Analysis of plasmids extracted from *E. coli* previously transformed with plasmid DNA from transconjugants. Plasmid DNA samples were digested with enzyme *Eco*RI (Fast Digest, Thermo Fisher Scientific), purified, and run on an agarose gel. pCOR48, plasmid DNA (positive control). The transconjugants from which the electroporated DNA was extracted were *L. casei* 393 (pCOR48) (1–2), and *S. epidermidis* (pCOR48) from two different colonies (3–4 and 5–6). M, molecular weight marker (GeneRuler 1 kb DNA ladder). The 6,000 and 3,000 bp are indicated. Expected fragment sizes: 5,436 and 2,000 bp.

The mobilizable shuttle plasmids used for our mating assays (pCOR48 and pCOR49; Table 2) carry origins of replication for *E. coli* and *Lactobacillus*, but it is not known if the plasmid can replicate in staphylococci. In order to determine if the *S. epidermidis* transconjugants harbored the shuttle plasmid as an episome, or they were the result of integration of the plasmid into the *S. epidermidis* chromosome, plasmid DNA was extracted from several transconjugants. In parallel, plasmid DNA was extracted from the strain without plasmids as a negative control, and from *L. casei 393* transconjugants obtained in previous conjugation assays, as positive controls. The DNA samples were run on agarose gels, where the plasmid DNA was visible in the *S. epidermidis* transconjugants (not shown). For further confirmation, these plasmid DNA samples were used to electroporate *E. coli.* Ampicillin-resistant colonies were obtained and their plasmid DNA was extracted and analyzed by restriction digestion in parallel with the original plasmid DNA, to confirm the presence of the pCOR shuttle plasmid (Figure 3). It can be observed that the plasmid recovered from the *E. coli* cells transformed with plasmid DNA extracted from the *L. casei* or *S. epidermidis* transconjugants maintains the same restriction pattern as the original shuttle plasmid present in the donor *E. coli* strain. Thus, the shuttle plasmid is able to replicate in *S. epidermidis*.

## Discussion

Targeted genetic modification of bacteria with biotechnological and biomedical potential is a prerequisite for most processes of genetic improvement, whether it is to introduce a plasmid contributing to increase production of the desired substance, or to introduce scarless genetic modifications in strains to be applied to human consumption or for medical uses. These processes all have a first requisite consisting on the introduction of DNA into the target cell. A number of protocols exist using bacterial transformation, conjugation, phage transduction, or even protoplast fusions, which are available for most laboratory strains. However, the need for novel substances to use as antimicrobials, food additives, probiotics, or therapeutic substances, has propelled the search for wild-type strains providing the desired properties, which require subsequent optimization steps. Introducing foreign DNA into these microorganisms often proves challenging and even impossible. Among LAB, electroporation is the most widely used method, due to its simplicity, efficiency and wide applicability; however, efficiencies vary strongly among species and even strains, and protocols need to be optimized for each of them (Wang et al., 2020). In particular, transformation of wild-type strains has proven difficult or even not feasible. This is the case for many lactobacilli which, in addition of including some of the most relevant species in the food industry, has an increasingly important biomedical interest, due to both its potential as human live delivery vector and to the existence of emerging human pathogens. Thus, there is an open niche for new DNA introduction protocols.

Bacterial conjugation is a naturally efficient and promiscuous mechanism of horizontal gene transfer, which operates among all main bacterial types. Conjugative DNA transfer from *E. coli* to several LAB has been reported (see section “Introduction”), but to our knowledge, there are no reports of conjugation from *E. coli* into lactobacilli. In this work, we prove that it is possible to introduce DNA by conjugation into lactobacilli from *E. coli*, not only to the model laboratory strain *L. casei* 393, but to a number of other genera, species and natural isolates, typically reluctant to transformation. In particular, we successfully obtained transconjugants in *L. plantarum, L. buchneri*, *L. parabuchneri, L. brevis, and L. vaginalis* (Table 6). Since no transconjugants were obtained in the negative controls lacking the conjugative machinery, we conclude that DNA transfer is happening through conjugation, and not through other mechanisms such as natural transformation, nanotubes, or extracellular vesicles. Dedicated electroporation protocols have been published for each of these species, reflecting the inherent difficulty of transforming them (Stephenson et al., 2011; Spath et al., 2012; Zhang et al., 2012). In the case of *L. parabuchneri*, to our knowledge there are no reports of transformation, which makes this result especially significative. *L. parabuchneri* is a member of cheese flora, contributing to its organoleptic properties and ripening process (Fröhlich-Wyder et al., 2013). Moreover, some species have been characterized as potential probiotics (Agostini et al., 2018). On the other hand, some strains of *L. parabuchneri* are mainly responsible for the undesirable accumulation of the biogenic amine histamine in cheese (Diaz et al., 2015b). Thus, the ability to manipulate genetically this species has high scientific interest, as well as both biotechnological and biomedical potential.

The efficiency of conjugation into the model laboratory strain *L. casei* 393 was around 10^–3^ or 10^–4^ transconjugants per donor, depending on the conjugative system used (RP4 or R388), which is higher than in early reports of conjugative transfer between distantly related bacteria (Trieu-Cuot et al., 1987). Comparable rates were obtained in conjugation experiments from *E. coli* to *Bifidobacterium* (Dominguez and Sullivan, 2013) where differences between strains were apparent. The efficiency of the different lactobacilli species as recipients also varied widely, and was always lower than that of the laboratory strain (Table 6). Some species were not transformed. There is no taxonomic explanation for this difference: according to the recent reclassification of the genus *Lactobacillus* (Zheng et al., 2020), *L. casei* shares the genus with its closest relative *L. paracasei*, for which we obtained no transconjugants. In fact, a survey of different *L. casei* natural isolates (Table 5) showed also ample variation within the species. The difference in conjugation frequencies could have multiple causes, such as the existence of different restriction-modification systems, and very likely, the presence of other plasmids in the wild-type strains; further studies would be necessary to determine the factors interfering with conjugation, which could lead to increased efficiencies and a wider range of potential recipients. Moreover, as for some species the frequency of conjugation obtained in some cases is just at the limit of detection of the mating assays, we think that an optimization of the protocol will probably extend the range of recipient species.

Serendipitously, we found that our conjugation assay also mediated DNA transfer into a researcher-contaminant *S. epidermidis*, so we isolated this species from different researchers and quantified the DNA transfer, confirming the presence of transconjugants. We have also confirmed the episomal presence of the shuttle plasmid in the transconjugants, meaning that one of the origins of replication present in this plasmid is functional in staphylococci. The mobilizable shuttle plasmids pCOR48 and pCOR49 contain the pBR322 origin of replication, which is functional in *E. coli* but not in Gram-positive organisms, and the replicon of P8014-2, a plasmid isolated from *L. plantarum* (Leer et al., 1992). There are a number of broad-host-range plasmids of Gram-positive bacteria which can replicate in both *Lactobacillus* and *Staphylococcus* spp. (Jain and Srivastava, 2013). Our results indicate that the *Lactobacillus* plasmid P8014-2 replicon is also functional in both lactobacilli and staphylococci. In fact, this replicon includes a sequence at position 2051 (5′-TTCTTATCTTGATA-3′) which is identical to the plus origin of replication of plasmid pC194 (Gros et al., 1987), capable of replication in *S. aureus* and *Bacillus subtilis* (Horinouchi and Weisblum, 1982).

Our finding that bacterial conjugation can be used to introduce DNA into *S. epidermidis* is significant. As stated in the Introduction, staphylococci are difficult to transform, and conjugative DNA transfer from *E. coli* has only been reported for *S. aureus* (Trieu-Cuot et al., 1987) using an IncP plasmid. *S. epidermidis* is a component of the human microbiota and also an emerging pathogen (Otto, 2009), leading to an increasing interest in its genetic manipulation. Up to now, few reports have addressed electroporation and transduction, respectively, of specific *S. epidermidis* strains (Monk et al., 2012; Winstel et al., 2015; Costa et al., 2017). The strategies used to increase the transformation efficiency of *S. aureus* have little efficiency on *S. epidermidis* (Monk et al., 2012; Costa et al., 2017). Thus, adding bacterial conjugation to this scarce toolbox will undoubtedly facilitate the generation of genetically modified strains. Conjugation into the laboratory strain *L. casei* 393 worked efficiently using both RP4 and R388 conjugative systems, although the efficiency was higher with the RP4 system. This result was expected, since the RP4 transfer system has been widely used to transfer DNA into distantly related bacteria and even eukaryotic cells, due to its intrinsic promiscuity (Bates et al., 1998; Luzhetskyy et al., 2006). In contrast, this is, to our knowledge, the first report of conjugative transfer to any Gram-positive bacteria mediated by R388. Moreover, conjugation into *S. epidermidis* was more efficient using R388 than RP4 conjugative system (Table 7). These results underscore the importance of assaying different conjugative systems, and point to the R388 conjugative system as a suitable candidate to explore other recalcitrant microorganisms as recipients of bacterial conjugation assays.

With this work, we show that a single conjugation protocol allows the introduction of foreign DNA into many different genera, species, and wild-type strains. The result obtained accidentally with *S. epidermidis* suggests that the range of Gram-positive bacteria which can act as recipients of conjugative DNA transfer from *E. coli* may be wider than suspected. Using *E. coli* as a donor laboratory strain implies access to almost unlimited genetic tools to generate the desired DNA to be transferred. Bacterial conjugation is a simple assay, which allows the transfer of DNA molecules of any size, even whole genomes (Isaacs et al., 2011). In addition, bacterial conjugation is considered a natural process, as opposed to electroporation; conjugation has been exploited to introduce natural plasmids into LAB strains, which can be considered non-genetically modified when this technology is used instead of electroporation (Pedersen et al., 2005; Bron et al., 2019). These features are relevant for the genetic manipulation of LAB, for their use in food fermentation as probiotics or as live vector for mucosal delivery of therapeutic proteins (Wells and Mercenier, 2008).

## Data Availability Statement

The original contributions presented in the study are included in the article/supplementary material, further inquiries can be directed to the corresponding author/s.

## Author Contributions

MÁ and ML conceived the work. SS, DG-H, RM-C, and MM performed the experiments. SS, DG-H, RM-C, MÁ, and ML analyzed the results. SS, MÁ, and ML wrote the manuscript. All authors contributed to the article and approved the submitted version.

## Conflict of Interest

The authors declare that the research was conducted in the absence of any commercial or financial relationships that could be construed as a potential conflict of interest.

## Dedication

This article is dedicated to the memory of María Cruz Martín.
